# Assessment of treatment efficacy of diphenylcyclopropenone (DPCP) for alopecia areata

**DOI:** 10.3906/sag-1807-230

**Published:** 2020-12-17

**Authors:** Zekayi KUTLUBAY, Ayşegül SEVİM KEÇİCİ, Övgü AYDIN, Suphi VEHİD, Server SERDAROĞLU

**Affiliations:** 1 Department of Dermatology, Cerrahpaşa Medical Faculty, İstanbul University-Cerrahpaşa, İstanbul Turkey; 2 Department of Dermatology, Haydarpaşa Numune Training and Research Hospital, University of Medical Sciences, İstanbul Turkey; 3 Department of Pathology, Cerrahpaşa Medical Faculty, İstanbul University-Cerrahpaşa, İstanbul Turkey; 4 Department of Biostatistics, Cerrahpaşa Medical Faculty, İstanbul University-Cerrahpaşa, İstanbul Turkey

**Keywords:** Alopecia areata, topical immunotherapy, diphenylcyclopropenone

## Abstract

**Background/aim:**

Alopecia areata (AA) is an inflammatory disease with a genetic and autoimmune basis. Herein, it was aimed to study the efficacy and safety of an immunomodulatory therapeutic agent, diphenylcyclopropenone, while manifesting its association with histopathological features, prognostic factors, and side effects.

**Materials and methods:**

In this retrospective study, 98 patients (60 males, 38 females) with alopecia, who were referred to the Hair Disease Polyclinic at the Department of Dermatology, between 2011 and 2015, were included. Together with medical histories and dermatological examinations, a skin biopsy for histopathological examination was conducted for all of the patients prior to therapy. Therapeutic success was evaluated on the basis of the hair regrowth percentage.

**Results:**

Regarding the overall treatment success, 33 (34%) patients had complete response, 16 (16%) had partial response (between 50% and 99%), 27 (28%) had minimal response (between 1% and 49%), and 22 (22%) were nonresponders. Both sexs were equally represented in the outcome.

**Conclusions:**

There was a significant relation between the severity of alopecia and the treatment outcome (P = 0.038). Patients with AA had significantly better response when compared to those with alopecia totalis and universalis. There was no statistically significant relation with other parameters, such as disease duration, age, sex, atopy history, age of onset, and histopathological features.

## 1. Introduction

Alopecia areata (AA) is an inflammatory disease with a genetic and autoimmune basis, leading to various degrees of nonscarring patchy hair loss of the scalp and whole body areas. The estimated prevalence of AA is between 0.1% and 0.2% [1]. The exact aetiology of AA is still unknown, but genetic disposition has been identified, with some genes like TRAF1/C5 locus. Autoimmunity also plays a major role, with accompanying diseases of T cell auto-reactivity, such as Hashimoto’s tiroiditis or vitiligo. According to some studies, having a history of atopy also increases the risk of AA [2]. Cytokines like interferon-gamma, interleukins, and tumor necrosis factor alpha are thought to play a major role in the pathogenesis of the disease.

Clinically, patients present with asymptomatic, patchy hair loss, with normal appearing underlying skin. Spontaneous resolution can occur, especially for those with limited involvement. However many patients have a chronic and recurrent course, with many attacks over the years. Treatment of alopecia has always been a big challenge for physicians. There are many different treatment modalities, with variable efficacies, including topical, intralesional or systemic corticosteroids, cyclosporine-A, local or systemic phototherapy, interferon-α, photodynamic therapy, acupuncture, topical minoxidil, anthralin, and topical immunotherapy agents.

Topical application of diphenylcyclopropenone (DPCP) was originally developed in 1978 by Happle et al. [3]. The exact mechanism of action of this treatment modality is not fully discovered. However, DPCP induces an allergic contact dermatitis and this is thought to decrease the T-cell-mediated immune reaction against the hair bulb. There is also evidence that it acts on the autoreactive T-lymphocytes within the follicular milieu to induce apoptosis. DPCP immunotherapy has a modulatory effect on the proinflammatory cytokines within the hair follicle, and also provides antigenic competition, to distract the lymphocytes from their primary target.

In this study, the efficacy and safety of topical immunotherapy with DPCP were evaluated retrospectively. The favorable prognostic factors to predict the response to DPCP immunotherapy were also assessed.

## 2. Material and methods

### 2.1. Patients and study design

In this retrospective study, 98 patients (60 males, 38 females) with alopecia areata, totalis, or universalis, who were referred to the Hair Disease Polyclinic at the Department of Dermatology of Cerrahpaşa Medical Faculty, from August 2011 to June 2015, were included. All of the patients had clinically diagnosed alopecia and for all of them, the diagnosis was confirmed with histopathological examination. The patients had been resistant to any other topical or systemic conventional therapies for at least 6 months. Prior to the therapy, the patients were informed about the efficacy, estimated duration, and possible side effects of the therapy, such as enlargement of the lymph nodes on the head and neck area; severe eczematous reactions, such as bullous eruptions or urticarial plaques limited to the scalp or extending to other body parts; fever, malaise and fatigue, especially within 2 days after the treatment; and sometimes anaphylaxis-like reactions. All of the patients gave their informed consent. If a patient was below 18 years of age, consent was taken from their family. The mean age of the patients was 23.5 years, ranging from 5 to 59 years. Exclusion criteria included current pregnancy or lactation, being a child under the age of 5, and having vitiligo, severe photosensitivity, or active systemic malignancies. Before initiation of the therapy, factors such as sex, duration of the disease, age of onset, age at the beginning of therapy, previous treatments, history of atopy, type and severity of hair loss (in forms of percentage), presence of ophiasis pattern, and eyebrow, eyelash, beard, and body hair involvement were recorded. On the first visit, a physical assessment was performed, with emphasis on grading the percentage of scalp involvement, type of alopecia, and nail involvement. Prior to the therapy, a skin biopsy of 4 mm in diameter was taken from all of the patients, from the affected site on the scalp and histopathological investigations were conducted. The following parameters were assessed during the histopathological investigations of the scalp biopsies: number of total follicular units, number of total hair follicles, number of follicular stelae, number of anagen, catagen and telogen hair follicles, degree of lymphocytic infiltration of the hair follicle, and degree of perifollicular fibrosis. The degree of fibrosis was evaluated numerically as 0: no fibrosis, 1: mild fibrosis, 2: moderate fibrosis, and 3: severe fibrosis. Lymphocytic infiltration was also assessed similarly, as 0: no lymphocytic infiltration; 1: mild lymphocytic infiltration, which represented the involvement of less than 10% of total number of hair follicular bulbus and the presence of less than 3 rows of lymphocytes around each hair follicle; 2: moderate lymphocytic infiltration, which represented involvement of 10%–50% of the hair follicular bulbus and the presence of 3 to 6 rows of lymphocytes around each hair follicle; and 3: severe lymphocytic infiltration, which represented the involvement of more than 50% of all hair follicular bulbus and the presence of more than 6 rows of lymphocytes around each hair follicle.

At the end of the study, therapeutic success was evaluated on the basis of the percentage of hair regrowth on the scalp.

### 2.2. Treatment method with DPCP

Topical immunotherapy with DPCP was performed following a standard protocol of sensitization. This protocol comprised the application of a 2% concentration of DPCP solution diluted in acetone, over an area of 2 × 2 cm on the occipital region of the scalp. The patients were told to avoid water contact of the sensitized area for 2 days and avoid sun exposure by using a wig or protective hat. After 2 days, the patients were checked to detect whether or not sensitization to DPCP had occurred. There was a lag period of 2 weeks after the sensitization, and then the treatment began. The initial DPCP concentration was 0.001%. DPCP solution was applied to all of the affected areas on the scalp, together with the eyebrows, once every week. Again after each session, the patients were told to avoid water contact for 2 days, including excessive sweating and sun exposure. DPCP solution was left on the scalp for 48 h and then washed off with a mild shampoo. The concentration of the DPCP solutions were increased weekly, unless there were serious side effects, including irritant contact dermatitis and photoallergic reactions, and the final concentration of 2% was reached at the end of week 6. Concentrations of 0.001%, 0.01%, 0.1%, 0.2%, 0.5%, 1%, and 2% were applied sequentially.

### 2.3. Follow-up and assessment of efficacy

During follow-up visits, the side effects of the patients were recorded, as well as the grade of hair regrowth. Once complete or cosmetically acceptable hair regrowth (amount of growth that eliminated the need for using a wig or hat) was achieved, the intervals of the DPCP application were prolonged to 2 weeks, 3 weeks, and monthly. By this method, the DPCP immunotherapy was discontinued gradually. In the case of hair loss during this tapering-off period, therapy was restored at weekly intervals. If there was no obvious response at the end of 6 months, immunotherapy was considered as noneffective and discontinued.

### 2.4. Statistical analysis

Statistical analyses were performed using SPSS 19.0 for Windows (SPSS Inc., Armonk, NY). The Mann–Whitney, Wilcoxon, and Kruskal-Wallis statistical tests were performed for each data set and P < 0.05 was considered as statistically significant.

## 3. Results

The mean age of the patients was 23.2 years, ranging from 5 to 59 years. The mean age of the male patients was 21.8 years, with a standard deviation of 12.4. The mean age of the female patients was 25.5 years, with a standard deviation of 15.6. The demographic and clinical data are shown in Table 1. The mean period of disease was 4.2 years before the DPCP treatment (Table 1).

**Table 1:** Demographic and clinical data.

Demographic data	
Number of patients	98
Sex (M/F)	M: 60 (61%)/F: 38 (39%)
Mean age	23.2 years (Male: 21.8, Female: 25.5)
Age at onset	Mean: 28 years, Range: 4–55 years
Disease duration	4.2 years
Previous local treatments	37 patients
Previous systemic treatments	61 patients
Mean treatment duration (months)	13.1
Mean treatment sessions	50.5
Clinical data	
Type of alopecia	
Alopecia areata	49
Alopecia totalis	19
Alopecia universalis	30

Among the 98 patients, 49 (50%) were diagnosed with AA, while 19 (19.4%) had alopecia totalis, and 30 (30.6%) had alopecia universalis. At the beginning of the immunotherapy, 9 (9.25%) of the patients had less than 25% hair loss, which according to the Alopecia Areata Investigational Assessment Guidelines, was categorized as S1, while 13 (13.25%) had between 25% and 50% (S2), 16 (16.25%) had between 50% and 75% (S3), and 11 (11.25%) had between 75% and 99% (S4). Of the patients, 49 (50%) had total hair loss on the scalp area (S5), with a diagnosis of alopecia totalis or universalis.

Additionally, 20 (20.4%) patients had a previous history of atopy, including atopic dermatitis or allergic asthma.

With regards to the previous treatments, 37 (37.7%) patients had used only topical treatment modalities, including topical corticosteroids, minoxidil (2% or 5% concentration), and intralesional corticosteroid injections. Of the patients, 61 (62.3%) had tried both topical and systemic treatment options, including systemic corticosteroids, cyclosporine, and ultraviolet therapy.

The mean duration of DPCP immunotherapy in the analyzed cohort was 13.1 months, with a minimum of 4 months and a maximum of 44 months.

**Figure 1:**
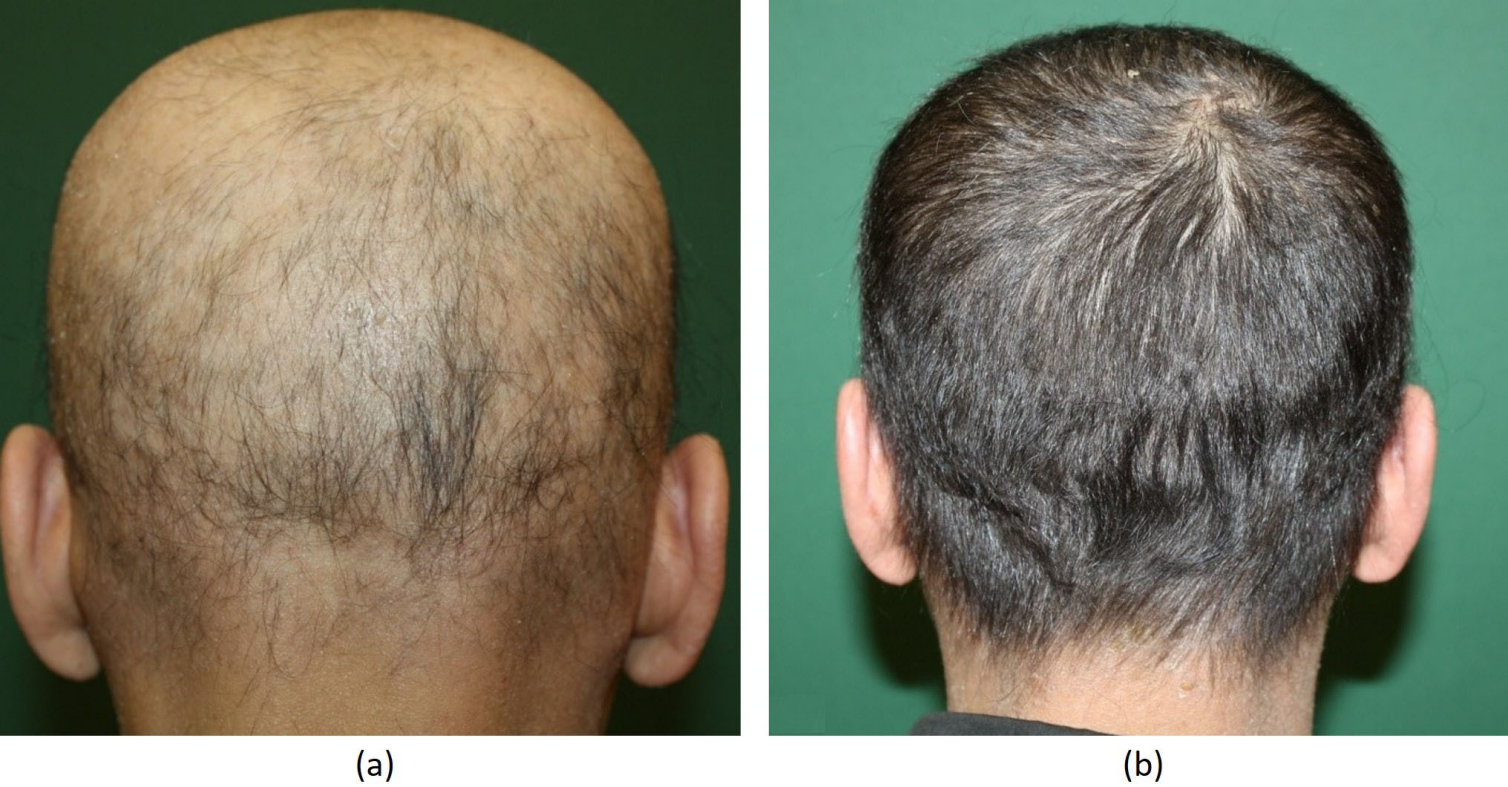
(A) Appearance of the patient before DPCP. (B) Full clinical response after 4 months of DPCP treatment.

**Figure 2:**
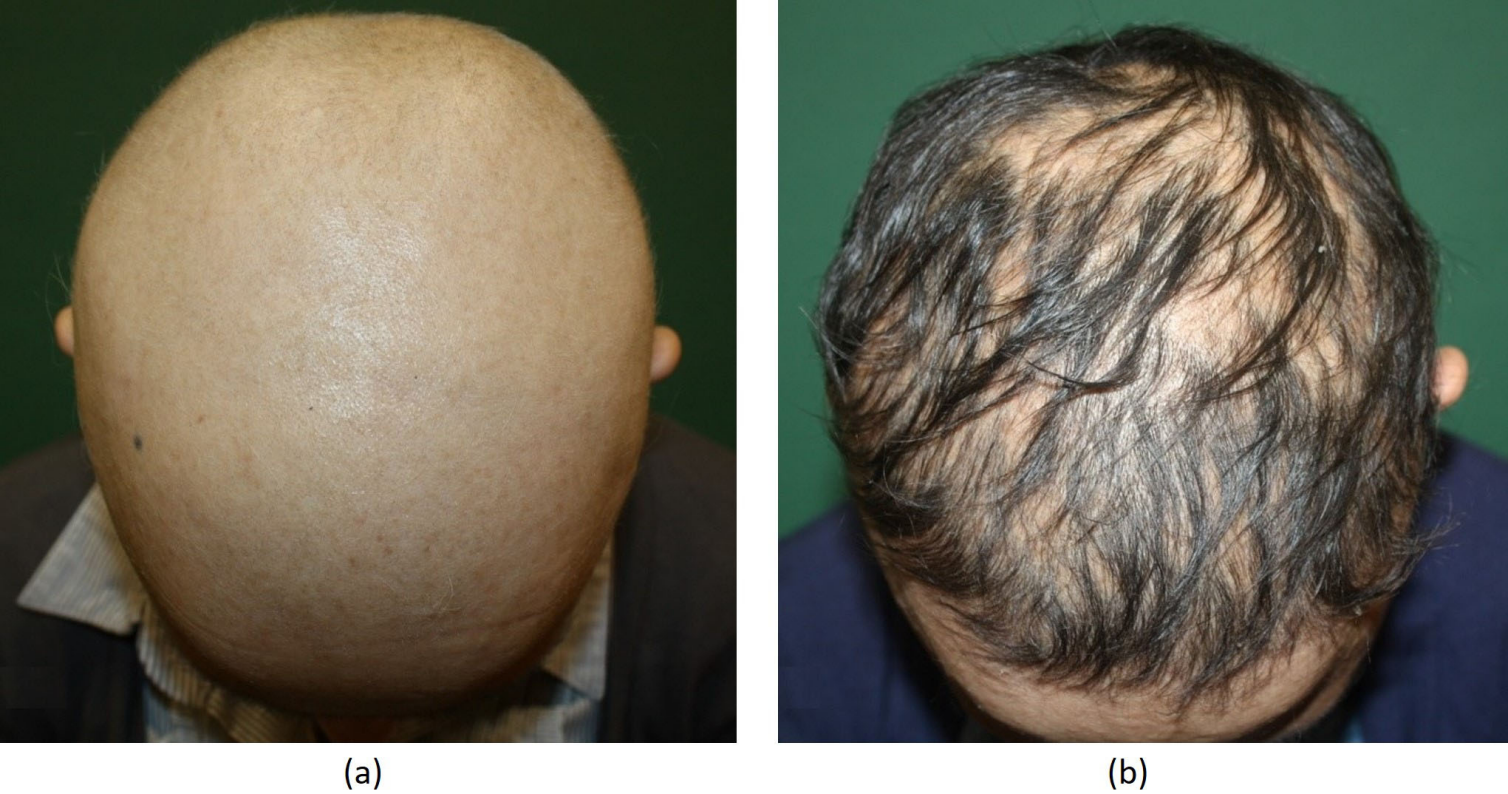
(A) Appearance of the patient before DPCP. (B) Cosmetically acceptable response after 7 months of DPCP treatment.

**Figure 3:**
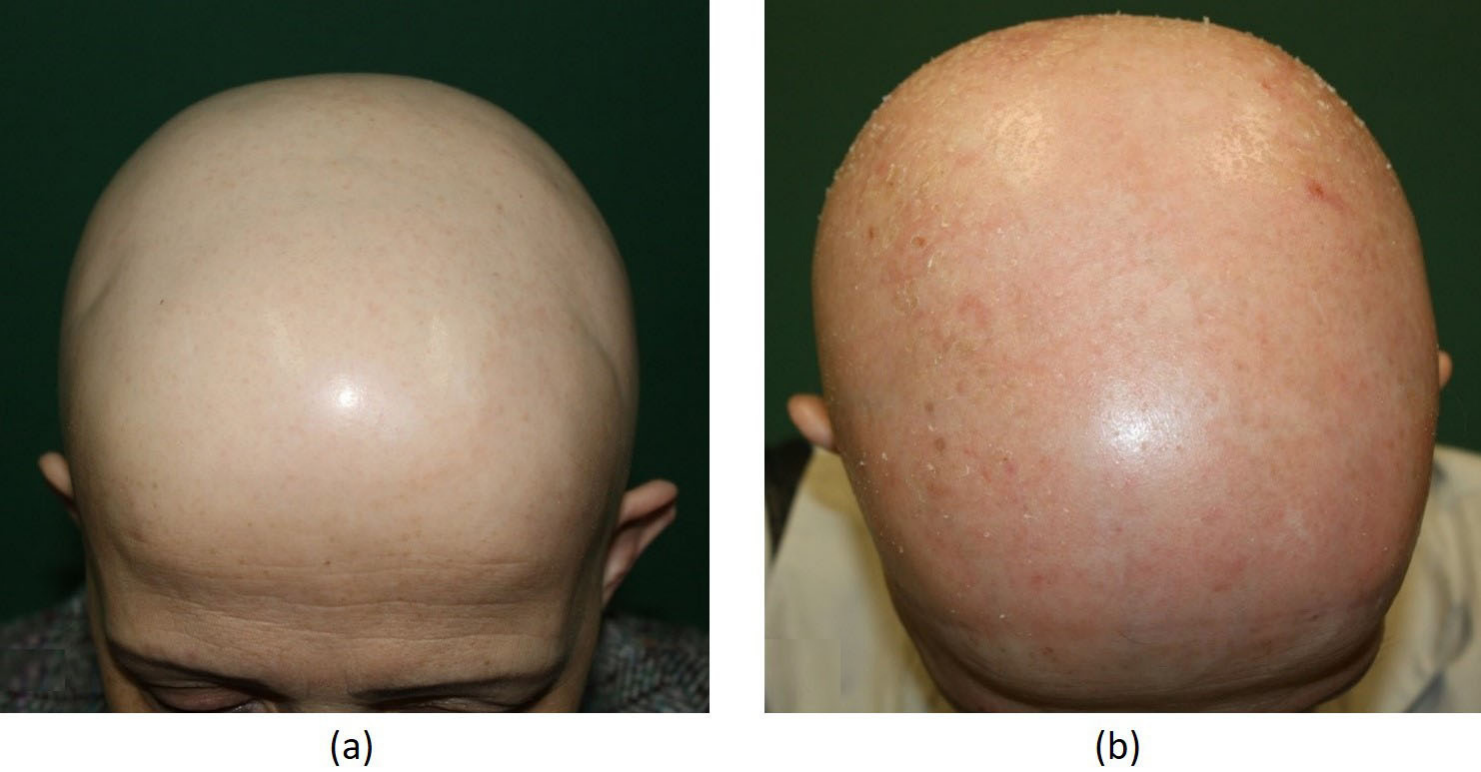
(A) Appearance of the patient before DPCP. (B) Patient not responding 6 months of DPCP treatment.

Regarding the overall treatment success, 33 (34%) patients had complete response (Figure 1A shows the pretreatment phase and Figure 1B shows complete response at 4 months posttreatment), 16 (16%) patients had partial response (between 50% and 99%) (Figure 2A shows the pretreatment phase and Figure 2B shows a cosmetically acceptable response at 7 months of treatment), 27 (28%) patients had minimal response (between 1% and 49%), and 22 (22%) patients were nonresponders (Figure 3A shows the pretreatment phase and Figure 3B shows the skin of the patient who did not respond to 6 months of treatment). Both sexes were equally represented in the outcome.

Among the 22 nonresponding patients, 8 responded by 100% and 7 responded by 75%–99% during the course of the therapy followed by a sudden and complete loss of hair.

The Mann–Whitney U test was used to investigate the relation between the type of alopecia and the treatment result. Statistical analyses revealed a significant relation between the type and severity of alopecia and the treatment outcome (P = 0.038). AA patients had significantly better response to the treatment when compared to the alopecia totalis or alopecia universalis patients. On the other hand, there was no statistically significant difference between the success rate of the therapy and the other parameters, such as the disease duration before DPCP therapy, age, sex, atopy history, age of onset of alopecia, and age at start of DPCP immunotherapy.

**Figure 4:**
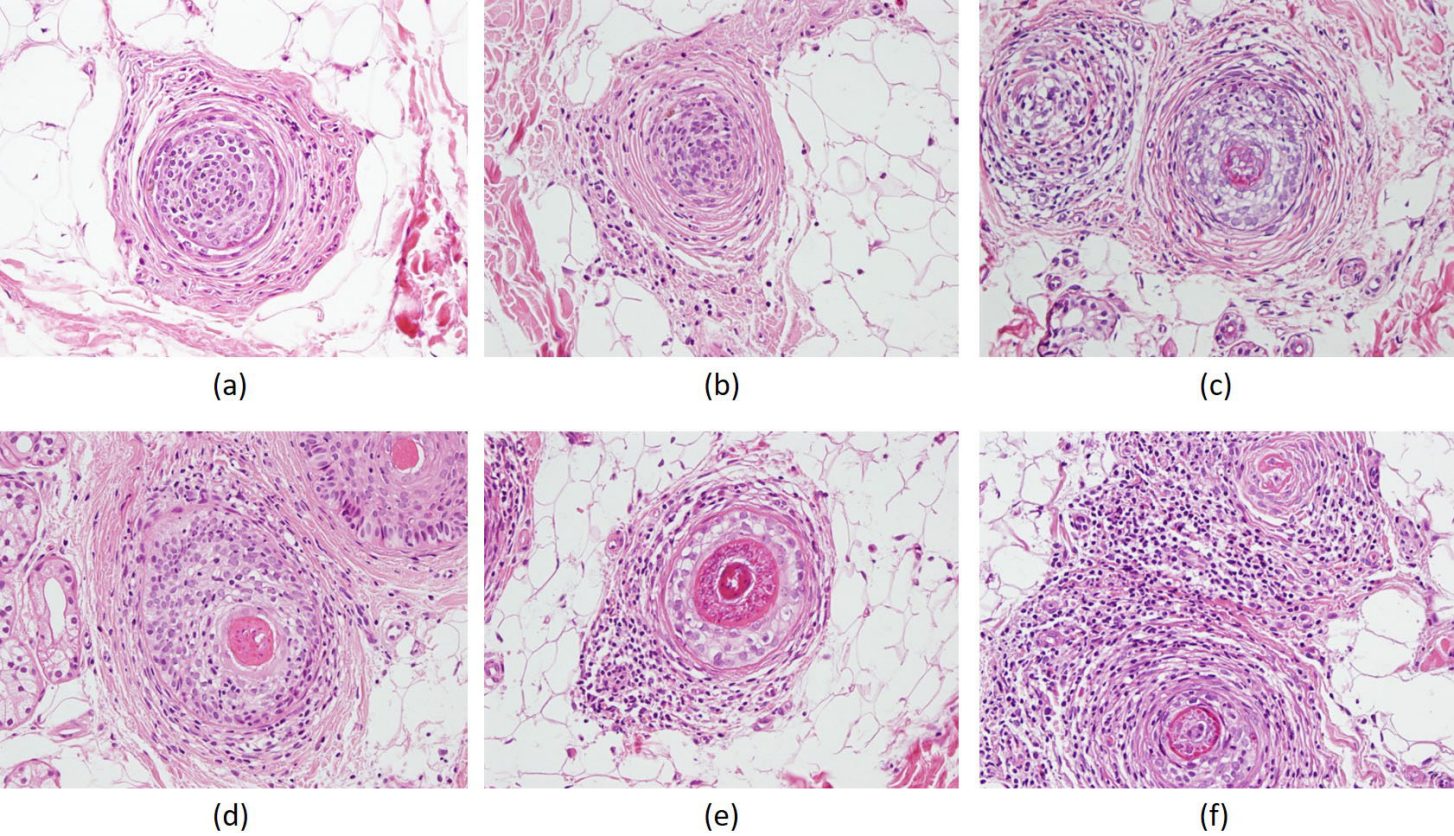
(A) Mild fibrosis, HEX400. (B) Moderate degree of fibrosis, HEX400. (C) Severe fibrosis, HEX400. (D) Mild degree of lymphocyte infiltration, HEX400. (E) Moderate degree of lymphocyte infiltration, HEX400. (F) Intense lymphocyte infiltration, HEX400.

Histopathological parameters were also compared with the success rates of the treatment, but there were no significant relationships with regards to the presence of fibrosis, lymphocytic infiltration, number of follicular stelae, and number of catagen hair follicles (Table 2; P = 0.478, P = 0.148, P = 0.994, and P = 0.118, respectively) Figure 4A shows mild fibrosis, Figure 4B shows moderate fibrosis, and Figure 4C shows severe fibrosis. In the case of lymphocyte infiltration, Figure 4D shows mild lymphocyte infiltration, Figure 4E shows moderate lymphocyte infiltration, and Figure 4F shows intense lymphocyte infiltration.

**Table 2:** Success rates and histopathological parameters with a comparison to the type of alopecia.

Type of Alopecia	Average success rate	P-value	Average lymphocytic infiltration*	Average fibrosis**
Alopecia areata (0%–25%)	74 %	0,012	2.7	1.4
Alopecia areata (25%–50%)	70 %	0.022	1.5	2.4
Alopecia areata (50%–75%)	67 %	0.034	1.6	1.9
Alopecia areata (75%–99%)	60 %	0.040	1.9	2.1
Alopecia totalis	55 %	0.045	2.1	2.0
Alopecia universalis	53 %	0.039	1.2	2.1

**Fibrosis is valued as 0: no fibrosis, 1: mild fibrosis 2: moderate fibrosis 3: severe fibrosis

The total number of sessions and thus, duration of therapy, that the patients received were found to be positively and significantly correlated with the success rates, meaning that the more sessions a patient received and the longer the therapy continued, and the better their response was.

During the sensitization process, almost all of the patients experienced minimal erythema, itching, and burning sensation over the affected area. However, during the course of the therapy, among the 98 patients, 56 (57%) showed no side effects and 38 (38%) complained about minimal erythema and sensitivity over the scalp, especially during the subsequent 2 days after the application. Other common side effects included pruritus, dermatitis, vesicles, bullae, and flu-like symptoms. Of the patients, 4 (4%) had serious side effects, such as lymph node enlargement, fever, and general malaise; 3 (3%) had irreversible hyperpigmentation of the head and neck area, and 2 (2%) developed vitiligo macules.

## 4. Discussion

AA is an organ-specific autoimmune disease, and the stem cells of the hair follicles are characteristically spared, which implies a potential for regrowth of the hair. Despite this fact, to date, there remains no curative treatment for AA and none of the existing treatment options have been able to alter the course of the disease. Phototherapy or photochemotherapy, topical and oral corticosteroids, and cyclosporine and biological agents, such as efalizumab and etanercept, have all been used with various success rates [4–8]. Among these treatment options, topical immunotherapy agents have revealed promising clinical success with low side effect profiles. Topical sensitizers, such as dinitrochlorobenzene, SADBE, or DPCP create an allergic contact dermatitis, via delayed-type (type IV) hypersensitivity reaction [9]. Dinitrochlorobenzene is a highly mutagenic and squaric acid dibutylester that can easily lose stability in the case of long-term use and high temperatures [10]. Thus, DPCP was the choice of topical agents for this study, as well as many others in the literature [11]. The exact mechanism of action for DPCP has still not been clarified; however, the likelihood of a regulatory-T lymphocyte mediated role within the follicular unit is high [12]. Although antigenic competition with the responsible antigens has been by far the most accepted theory, other remarkable effects over the cytokine system have included increased IL-10 secretion and low CD4+ to CD8+ T cell ratios, together with the induction of other immunomodulatory substances, such as CTLA4 [13,14].

In many studies of DPCP topical immunotherapy, initially, the solution was applied to one half of the scalp and only after there was hair growth, was the treatment extended to the entire scalp [15]. This approach aimed to determine the efficiency of the treatment while minimizing the side effects. However in the current study, after the sensitization process, the solution was applied to all of the hairless areas of the scalp, so as to obtain cosmetically acceptable results sooner.

In many studies, it was reported that combination therapy with minoxidil or other agents did not improve the success rates [16–18]. Therefore, any topical or systemic treatment other than DPCP was not given to the patient group.

The studies have revealed a wide range of treatment efficacies, with a success rate of up to 85% for cosmetically acceptable hair regrowth. In a study of 148 patients, 100% regrowth rate was achieved for patients with less than 50% hair loss [19–24]. This wide range of results could have been due to differences in the treatment protocols, data sets, evaluation methodology, prognostic factors, absence of uniform terminology for evaluating the results, and differences between the statistical methods used for analyzing the results. Some studies, on the other hand, have shown no differences between topical immunotherapy and placebo or other treatment modalities, such as topical corticosteroids, especially for patients with limited alopecia lesions [25,26]. These findings contradicted the previous data in the literature, as well as the current study, which clearly showed a better response in cases with limited involvement. According to the overall results of the studies in the literature, DPCP immunotherapy for AA is a powerful option with a limited side effect profile.

According to the previous efficacy studies of DPCP, chronic and extensive disease, and accompanying nail changes were reported to be poor prognostic factors [20], while limited involvement for a short period of time, absence of nail changes, older age of onset, and a negative history of atopic dermatitis correlated with a more favorable prognosis [27,28].

In the case series herein, the success rate and treatment response were only associated with the extent of hair loss and thus, the type of alopecia. Patients with limited AA had better prognosis and good response to therapy, while extensively involved alopecia totalis and alopecia universalis cases had worse a prognosis and resistant course. Other parameters, such as sex, duration of the disease, atopy history, age of onset of alopecia, and age at start of DPCP immunotherapy had basically no effect on the prognosis of the disease during DPCP immunotherapy. Many other studies have also supported this finding, with the severity of alopecia being the sole important factor for the outcome of the treatment [21,22].

Many adverse events have been reported due to the use of DPCP, including eczematous reactions, urticaria, vitiligo, lymphadenopathy, hyperpigmentation, or erythema multiforme-like reactions [18]. In the current study, during the sensitization process, almost all of the patients experienced minimal erythema, itching, and burning sensation. Overall, the most commonly encountered side effect was erythema and itching, followed by the formation of papules, vesicles, bullae, and flu-like symptoms. Other less frequent side effects were lymph node enlargement, fever, general malaise, irreversible hyperpigmentation of the head and neck area, and vitiligo macules.

The use of DPCP in children is still a controversial area, although some studies have shown good results with acceptable side effect profiles [29]. In the current study, children above 5 years of age were also included and the treatment results or safety parameters were similar to those of the adult patients.

AA can have serious aesthetic and psychological consequences for patients due to its unpredictable course, consisting of spontaneous remissions and relapses. All of the conventional treatment options have a limited chance of success. Topical DPCP immunotherapy is a safe and effective alternative for patients with resistant disease and long-term results will provide more precise data about the success rate of this treatment modality.

## Informed consent

The study protocol received institutional review board approval from ethical committee of the Cerrahpaşa Medical Faculty and all participants provided informed consent.
